# Regulation of mTOR Signaling by Semaphorin 3F-Neuropilin 2 Interactions *In Vitro* and *In Vivo*

**DOI:** 10.1038/srep11789

**Published:** 2015-07-09

**Authors:** Hironao Nakayama, Sarah Bruneau, Nora Kochupurakkal, Silvia Coma, David M. Briscoe, Michael Klagsbrun

**Affiliations:** 1 Vascular Biology Program, Boston Children’s Hospital and Harvard Medical School, 300 Longwood Avenue, Boston, MA 02115; 2 Transplant Research Program, Boston Children’s Hospital and Harvard Medical School, 300 Longwood Avenue, Boston, MA 02115; 3 Department of Surgery, Boston Children’s Hospital and Harvard Medical School, 300 Longwood Avenue, Boston, MA 02115; 4 Division of Cell Growth and Tumor Regulation, Proteo-Science Center, Ehime University, Toon, Ehime 791-0295, Japan; 5 Department of Pediatrics, Boston Children’s Hospital and Harvard Medical School, 300 Longwood Avenue, Boston, MA 02115; 6 Department of Pathology, Boston Children’s Hospital and Harvard Medical School, 300 Longwood Avenue, Boston, MA 02115.

## Abstract

Semaphorin 3F (SEMA3F) provides neuronal guidance cues via its ability to bind neuropilin 2 (NRP2) and Plexin A family molecules. Recent studies indicate that SEMA3F has biological effects in other cell types, however its mechanism(s) of function is poorly understood. Here, we analyze SEMA3F-NRP2 signaling responses in human endothelial, T cell and tumor cells using phosphokinase arrays, immunoprecipitation and Western blot analyses. Consistently, SEMA3F inhibits PI-3K and Akt activity, and responses are associated with the disruption of mTOR/rictor assembly and mTOR-dependent activation of the RhoA GTPase. We also find that the expression of vascular endothelial growth factor, as well as mTOR-inducible cellular activation responses and cytoskeleton stability are inhibited by SEMA3F-NRP2 interactions *in vitro*. *In vivo*, local and systemic overproduction of SEMA3F reduces tumor growth in NRP2-expressing xenografts. Taken together, SEMA3F regulates mTOR signaling in diverse human cell types, suggesting that it has broad therapeutic implications.

Neuronal networking is regulated by the response of axonal growth cones to environmental cues, both positive and negative. For instance, cues elicited by netrin-1 are chemoattractive, whereas those dominated by semaphorin 3F (SEMA3F) are chemorepulsive. These processes, known collectively as axon guidance, play an important role in the development of the central nervous system^1^,^2^. SEMA3F is a member of the class 3 semaphorins (SEMA3A-G), whose receptors are neuropilin 1 (NRP1), neuropilin 2 (NRP2) and Plexins^3^,^4^. Recently, we have demonstrated that semaphorins are involved in vascular and tumor biology^5^,^6^ and an increasing body of data indicate that they regulate the immune response pertinent to tumor immunity^7^,^8^,^9^,^10^. In addition, they inhibit the migration of endothelial cells (EC) and tumor cells *in vitro* and attenuate tumor progression, metastasis and angiogenesis *in vivo*^5^,^6^. Nevertheless, the response of T cells, EC, smooth muscle cells and tumor cells to SEMA3F is poorly understood but functional effects are characterized by regulatory responses including anti-migration, cytoskeleton collapse and loss of stress fibers^5^,^6^,^11^.

Our initial analysis of SEMA3F signaling mechanisms demonstrated that SEMA3F forms a complex with NRP2 and Plexin A1. This complex attracts the ABL2 tyrosine kinase, which activates p190RhoGAP, resulting in the inactivation of RhoA, a small GTPase that converts GTP to GDP, leading to depolymerization of F-actin and the loss of stress fibers with an associated diminished EC and tumor cell migratory response^6^. Gleevec (imatinib), an ABL2 tyrosine kinase inhibitor abrogates SEMA3-mediated loss of stress fiber formation and motility in glioblastoma cells and EC^12^. H157 lung cancer cells stably transfected with SEMA3F have reduced levels of phosphorylated Akt (S473), STAT3 and Erk^13^, and reduced Akt activity was associated with lower levels of expression of the angiogenic factor, vascular endothelial growth factor (VEGF)^13^.

While semaphorins and NRP-elicited responses may regulate multiple intracellular signaling pathways, a common feature is the inhibition of the phosphorylation of the Akt kinase^2^,^13^. This effect is highly suggestive that a major biological effect of semaphorin-induced signaling involves the inhibition of mTOR signaling. Indeed, a recent study demonstrated that invertebrate semaphorin-plexin interactions may regulate TOR signaling in *Caenorhabditis elegans* (*C. elegans*), which is required for morphological changes in its epidermal cells^14^. mTOR is a serine/threonine kinase that exists as two distinct multiprotein complexes, composed of either mTOR, raptor and mLST8 (mTORC1)^15^,^16^, or mTOR, rictor, Sin1, protor and mLST8 (mTORC2)^17^,^18^,^19^. mTORC1 controls cell growth in part by phosphorylating S6K1 and 4EBP1 and is a key regulator of protein translation^20^,^21^. mTORC2 mediates cell survival and activation by phosphorylating Akt^22^, serum/glucocorticoid-regulated kinase-1 (SGK1) and protein kinase C (PKC)α^18^,^23^. There is great interest in targeting mTOR signaling pathways as a therapeutic for autoimmune disease, chronic inflammation and allograft rejection^24^,^25^,^26^ and as an adjunct to cancer therapy^27^. Nevertheless, little is reported on the effects of SEMA3F on this signaling pathway or in these disease processes despite widespread expression of its NRP2 receptor on human immune, epithelial and tumor cells^5^,^6^,^28^.

In this report, we show that SEMA3F interacts with NRP2 and Plexin A1 to reduce PI-3K activity, inhibit the assembly of mTORC2 and reduce downstream Akt signaling. We also show that SEMA3F may elicit anti-tumor and anti-angiogenic effects by inhibiting PI-3K activity and Akt-induced transactivation of VEGF, which is well established to function in tumorigenesis and chronic inflammation. Collectively, our studies define SEMA3F as a novel PI-3K/mTORC2 inhibitor in mammalian cells, indicating that it has broad biological effects and potential as a therapeutic to enhance the resolution of chronic disease.

## Results

### SEMA3F inhibits Akt, mTOR, and S6K phosphorylation

**Figure 1:**
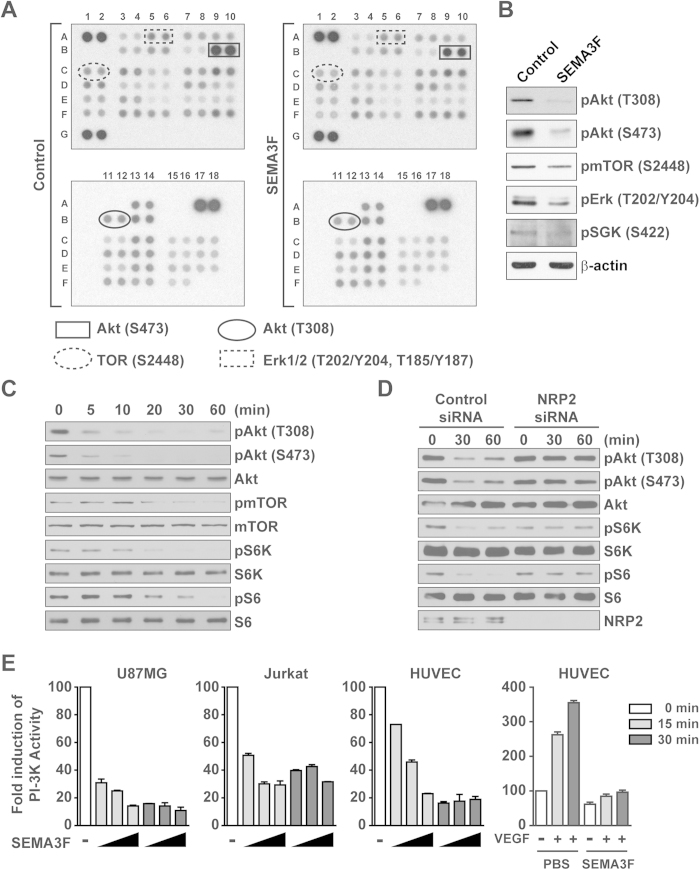
SEMA3F inhibits the phosphorylation of Akt, mTOR and S6K. **A**, U87MG cells untreated (control) or following treatment with SEMA3F (640 ng/ml) for 30 minutes. Cell lysates were evaluated by phosphoprotein kinase antibody array. The intensity of each dot/phosphoprotein was measured using Image J software, as shown in Supplementary Table 1. **B**, Results of the array were validated by Western blot analysis. **C**, U87MG cells were treated with SEMA3F (640 ng/ml) as a time course up to 60 minutes and were analyzed by Western blot. **D**, U87MG cells were transfected with a control siRNA or with a NRP2-specific siRNA (20 nM). After 48 hours, the cells were treated with SEMA3F (640 ng/ml) for 30 and 60 minutes and analyzed by Western blot. Panels **B–D** are representative of 3 independent experiments. **E**, U87MG, Jurkat and HUVEC cells were treated with SEMA3F (200, 600, 1800 ng/ml, bars from left to right) for 15 minutes (grey bars) or 30 minutes (black bars); as a positive control, HUVEC were treated with VEGF-A (25 ng/ml) for 15 and 30 minutes. In addition, HUVEC were pre-treated with SEMA3F (1800 ng/ml) or PBS as a control for 30 minutes and subsequently VEGF-A (25 ng/ml) was added to the culture for 15 and 30 minutes. PI-3K activity was analyzed by ELISA according to the manufacturer’s instructions. Data represent the mean ± SD of 3 experiments.

To determine the effect of SEMA3F on intracellular signaling responses, we initially profiled levels of phosphokinases in the NRP2-expressing human glioblastoma cell line U87MG. We found that SEMA3F inhibited the phosphorylation of a number of kinases, notably, Akt (T308 and S473), Erk (T202/Y204 and T185/Y187) and mTOR (S2448) (Fig. 1A and Supplementary Table 1), which was confirmed by Western blot analysis (Fig. 1B). A time course analysis further indicated that pAkt (T308 and S473), pmTOR and its downstream signaling (pS6K and pS6) were inhibited within 10–20 minutes of SEMA3F treatment, and this inhibitory effect persisted for greater than 60 minutes (Fig. 1C and Supplementary Fig. 1A). SEMA3F failed to inhibit pAkt, pS6K and pS6 in both NRP2 and Plexin A1-siRNA transfected cells, indicating that the regulatory effect of SEMA3F on Akt/mTOR activity requires interaction with NRP2/Plexin A1 complexes at the cell surface (Fig. 1D and Supplementary Fig. 1B). SEMA3F also inhibited Akt (S473) and S6K phosphorylation in several other cell lines expressing NRP2, including U251 glioblastoma cells, a melanocyte cell line, Jurkat T lymphocytes, and endothelial cells (Supplementary Fig. 1C,D). Using a standard ELISA-based assay^29^, we found that SEMA3F inhibited PI-3K activity in each cell line in a time dependent manner (Fig. 1E). In addition, pre-treatment of endothelial cells with SEMA3F (for 30 minutes) inhibited subsequent VEGF-induced PI-3K activation (Fig. 1E). These results indicate that SEMA3F-NRP2 interactions are regulatory to inhibit the activity of PI-3K-Akt/mTOR signaling.

### SEMA3F primarily inhibits the assembly of mTORC2

**Figure 2:**
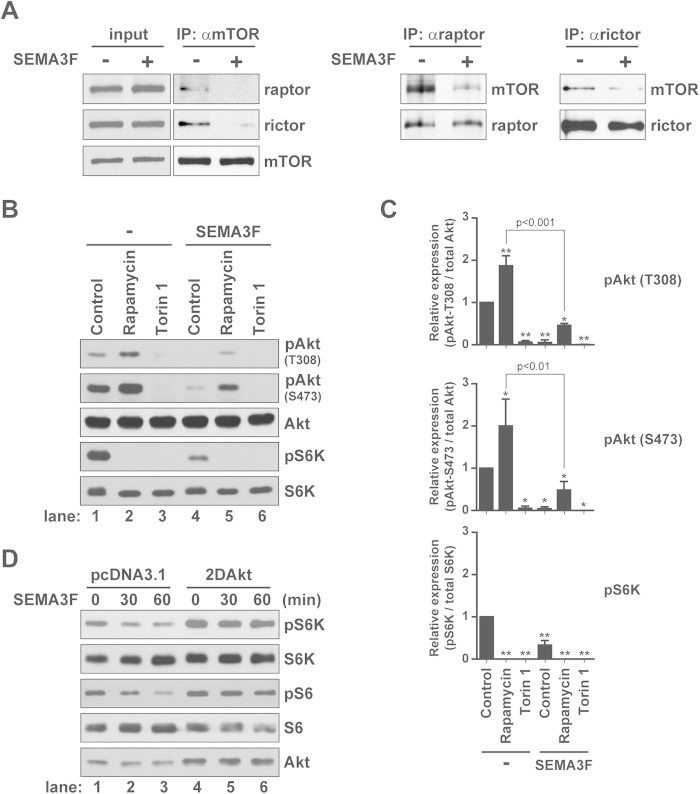
SEMA3F disrupts both mTORC1 and mTORC2 complex formation. **A**, U87MG cells were treated with SEMA3F (640 ng/ml) for 30 minutes and were subjected to immunoprecipitation and Western blot analysis with anti-mTOR, -raptor and -rictor as illustrated. **B**, U87MG cells were treated with rapamycin (10 nM) or Torin 1 (10 nM) for 30 minutes, prior to SEMA3F (640 ng/ml) treatment for 60 minutes; lysates were analyzed by Western blot. **C**, The right bar graphs represent densitometric analysis of the illustrated blot showing the fold change in intensity (mean ± SD) relative to the untreated control (*p < 0.01; **p < 0.001 vs. untreated control). **D**, U87MG cells were transiently transfected with a pcDNA3.1 empty vector or with constitutively active Akt (2DAkt). Cells were treated with SEMA3F (640 ng/ml) and lysates were analyzed by Western blot. All data are representative of 3 independent experiments.

mTORC1 and mTORC2 signaling is critical for cell metabolism^30^,^31^ as well as the differentiation, proliferation and survival of many normal cell types^27^,^32^,^33^,^34^,^35^. By immunoprecipitation, we found that SEMA3F inhibited the association between mTOR and both raptor and rictor (Fig. 2A), suggesting a biological effect on both mTORC1 and mTORC2 respectively. Consistent with this observation, cells treated with SEMA3F for 60 minutes had reduced levels of pAkt (T308 and S473) and pS6K (vs. untreated cells, Fig. 2B, lane 1 vs. 4). To determine if its primary mode of function relates to the inhibition of mTORC1 vs. mTORC2, we initially pretreated cells with rapamycin (10 nM for 30 minutes to inhibit mTORC1) and subsequently cultured cells in the absence or presence of SEMA3F and rapamycin for another 60 minutes. Treatment with rapamycin alone (for 90 minutes) as a control resulted in a marked inhibition of pS6K, but an induction in the levels of pAkt (T308 and S473) by 1.9-fold (p < 0.001) and 2.0-fold (p < 0.01), respectively (Fig. 2B, lanes 1 vs. 2 and Fig. 2C), as we and others previously reported^36^,^37^,^38^. In contrast, U87MG cells that were treated with rapamycin for 30 minutes and subsequently treated with SEMA3F and rapamycin for an additional 60 minutes had reduced levels of both pS6K and pAkt (Fig. 2B, lane 2 vs. 5). Of note, this effect of SEMA3F on the inhibition of pAkt expression was similar to that observed when cells are treated with the mTORC1/C2 inhibitor Torin 1 (Fig. 2B, lane 3 vs. 4). SEMA3F also inhibited pSGK1 (S422) and pPKCα (S657, Fig. 1B and Supplementary Fig. 2A), other known targets of mTORC2 activity^23^.

To further evaluate whether the primary target of SEMA3F is the mTORC2 complex, U87MG cells were transfected with 2DAkt, in which the T308 and S473 sites are mutated to encode a constitutively active form of the kinase^36^,^37^. Previously, we reported that overexpression of 2DAkt in EC resulted in mTORC1 activation/signaling, which was inhibited by rapamycin^37^. Similarly, transfection of 2DAkt was associated with induced levels of expression of pS6K and pS6 in U87MG cells vs. control transfectants (Fig. 2D, lane 1 vs. 4), but there was no change in expression following treatment of transfected cells with SEMA3F. However, SEMA3F reduced the level of pAkt (S473) in 2DAkt transfectants (Supplementary Fig. 2A) and by immunoprecipitation, the treatment of 2DAkt-transfected cells with SEMA3F inhibited mTOR/rictor interaction (Supplementary Fig. 2B). Together, these results demonstrate that SEMA3F/NRP2/Plexin A1 interactions have a direct effect on mTORC2 assembly which results in the inhibition of mTORC2/Akt activity.

### mTORC2 links SEMA3F biology with the F-actin cytoskeleton

**Figure 3:**
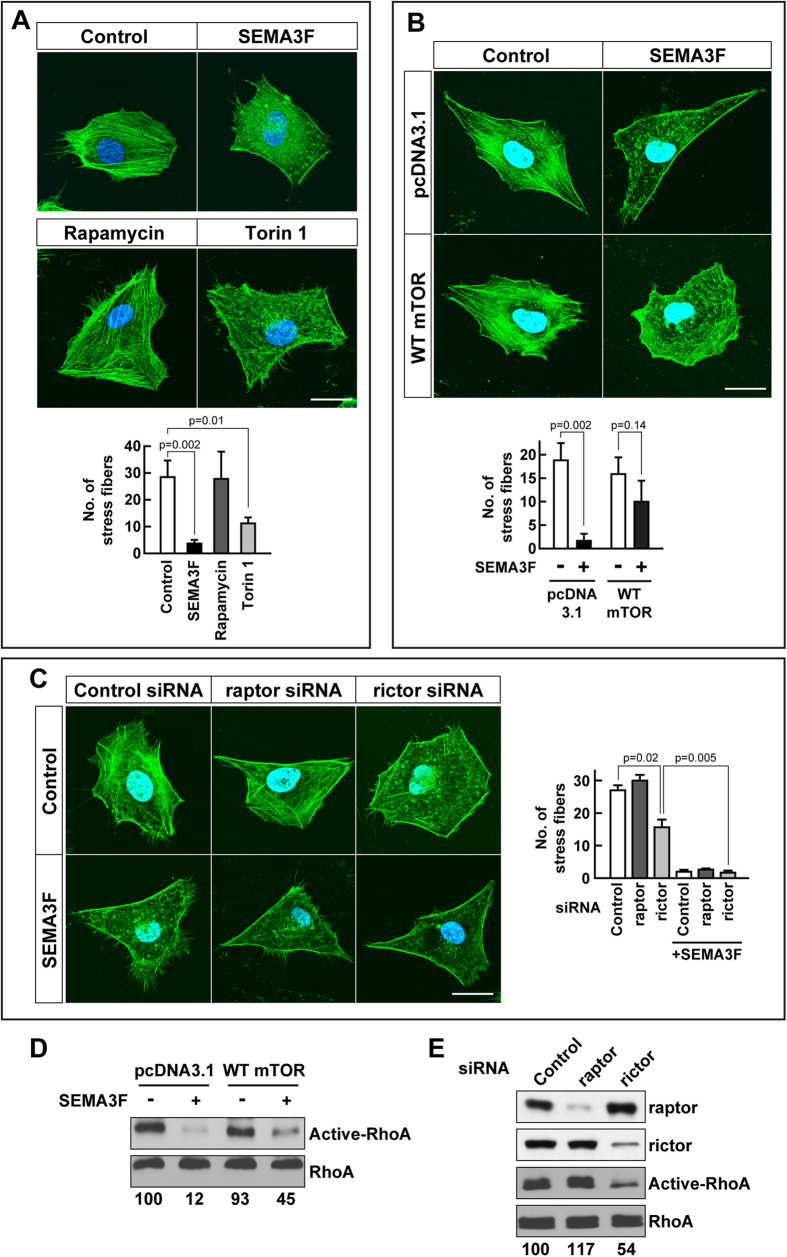
**mTORC2 participates in SEMA3F-induced RhoA inactivation and loss of stress fibers. A**, U87MG cells were treated with SEMA3F (640 ng/ml), rapamycin (10 nM) or Torin 1 (10 nM) for 30 minutes. Subsequently, cells were stained with Alexa Fluor 488 phalloidin and Hoechst 33342 to identify F-actin cytoskeleton stress fibers and cellular nuclei, respectively. Representative cellular staining of is shown in each panel; the bar graph shows the mean ± SD number of fibers/cell in an average of 3 independent experiments. The scale bar indicates 20 μm. **B**, U87MG cells were transiently transfected with a pcDNA3.1 empty vector or with a wild type (WT) mTOR plasmid and after 18 hours were treated with SEMA3F (640 ng/ml) for 30 minutes. Cells were stained as described above in A. Representative cellular staining is shown; bar graph represents the number of fibers/cell (mean ± SD) from 3 independent experiments. **C**, U87MG cells were transfected with control siRNA or with raptor- or rictor-specific siRNAs (20 nM). After 48 hours, they were treated with SEMA3F for 30 minutes and stained with Alexa Fluor 488 phalloidin and Hoechst 33342 as above. The number of stress fibers was evaluated in 3 independent experiments and shown as the mean ± SD. **D**, U87MG cells were transiently transfected with pcDNA3.1 empty vector or with our WT mTOR plasmid. After 18 hours, the cells were treated with SEMA3F (640 ng/ml) for 10 minutes and RhoA activity was evaluated. **E**, U87MG cells were transfected with control siRNA or with raptor- or rictor-specific siRNAs (20 nM), were treated with SEMA3F (640 ng/ml) for 10 minutes and RhoA activity was analyzed. In Panels **D**–**E**, the intensity of active RhoA was normalized to respective total RhoA; the numbers below each gel lane represent the fold-change in intensity relative to control. Panels **D**–**E** are representative of 3 independent experiments.

We next wished to determine whether the inhibition of mTORC2 serves as an intermediary response to link SEMA3F activity with cytoskeletal collapse. U87MG cells were treated either with SEMA3F, rapamycin or Torin 1 for 30 minutes and the actin cytoskeleton was visualized using phalloidin staining. As we previously reported^6^, SEMA3F markedly inhibited stress fiber formation and cytoskeletal arrangement compared to untreated cells (Fig. 3A, p = 0.002). Moreover, a similar effect was observed in cells treated with the mTORC1/C2 inhibitor Torin 1 (p = 0.01). In contrast, treatment with the mTORC1 inhibitor rapamycin failed to elicit any cytoskeletal changes (Fig. 3A; Supplementary Fig. 3). Also, while SEMA3F inhibited stress fiber formation by 90% in pcDNA3.1 control vector transfected cells, it had minimal effects in U87MG cells transfected with an mTOR overexpression construct (Fig. 3B). We interpret these findings to suggest that the effect of SEMA3F on stress fiber formation is not mediated via mTORC1. Consistent with this interpretation, knockdown of raptor also had minimal effects on stress fiber formation and cytoskeleton collapse (Fig. 3C). However, SEMA3F reduced stress fiber formation in raptor-siRNA treated cells (Fig. 3C); and notably, siRNA knockdown of rictor alone was sufficient to elicit collapse (p = 0.02). These data suggest that mTORC2 serves as an intermediary to modulate SEMA3F-inducible cytoskeletal collapse.

We next measured RhoA activity^39^ using the rhotekin pulldown assay in U87MG cells transfected with the mTOR overexpression construct (Fig. 3D). Consistent with our previous findings^6^, RhoA activity was suppressed by SEMA3F in control pcDNA3.1-transfected cells (by 88%), but activity was partially rescued in cells overexpressing mTOR (by 55%). In addition, using siRNAs (as above) we found that knockdown of rictor, but not raptor, attenuated RhoA activity (Fig. 3E). Collectively, these observations demonstrate that the inhibition of mTORC2 activity by SEMA3F/NRP2/Plexin A1 interactions is functional to inactivate RhoA, which in turn leads to cytoskeleton collapse.

### SEMA3F inhibits hypoxia-induced production of VEGF via the mTOR pathway

**Figure 4:**
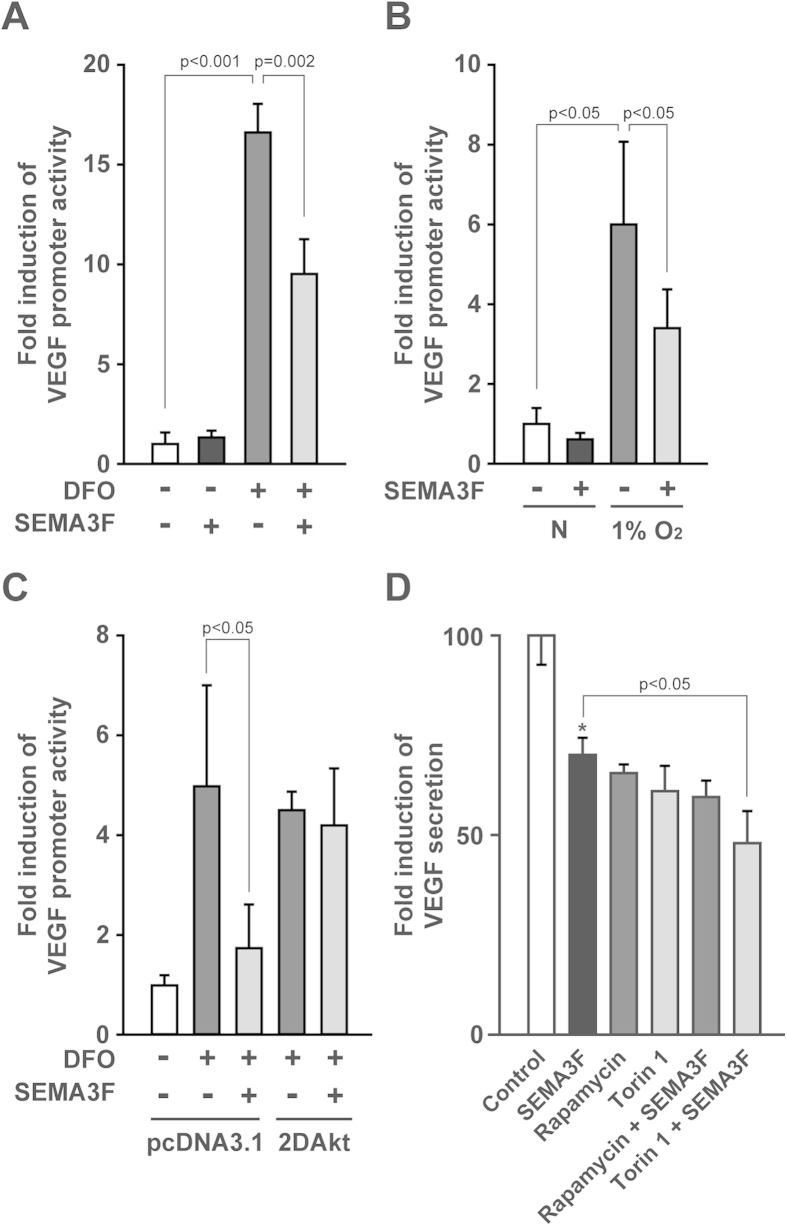
SEMA3F suppresses VEGF through the inhibition of mTOR-Akt signals . **A,B**, U87MG cells were transiently co-transfected with a full-length human VEGF promoter luciferase plasmid and a pGL4.74[hRluc/TK] plasmid as an internal control. Cells were treated with SEMA3F (640 ng/ml for 30 minutes) prior to the addition of DFO (250 μM) or the culture of cells in a hypoxia chamber (1% O_2_). After 18 hours, VEGF promoter luciferase activity was analyzed. **C**, U87MG cells were transiently co-transfected with our VEGF promoter luciferase and pGL4.74[hRluc/TK] plasmids and with either a pcDNA3.1 empty vector or our constitutively active Akt (2DAkt). The cells were treated with SEMA3F for 30 minutes prior to the addition of DFO. After 18 hours, VEGF promoter luciferase activity was analyzed. **D**, Parental U87MG cells were treated with SEMA3F, rapamycin (10 nM), Torin 1 (10 nM) alone or in combination as indicated for 30 minutes prior to the addition of DFO, and culture supernatants were collected after 18 hours; VEGF protein levels were analyzed by ELISA. In each panel data are representative of 3 independent experiments. Bar graphs represent the mean ± SD of n = 3 experiments performed in triplicate, *p < 0.01 vs. control.

The local expression and regulation of VEGF is key to many physiological and pathological processes^40^,^41^. Our previous findings are suggestive that SEMA3F may also target the transcriptional activation of VEGF^36^,^42^. To test the effect of SEMA3F on the regulation of VEGF expression, U87MG cells were transfected with a full-length 2.6 kb VEGF promoter-luciferase construct and exposed to the hypoxia mimetic agent desferrioxamine (DFO) or hypoxia (1% O_2_). We found that treatment with DFO induced VEGF promoter activity (by 16-fold, p < 0.001), which was partially inhibited (43%, p < 0.005) by SEMA3F (pre-treatment for 30 minutes, Fig. 4A). VEGF promoter activity was also increased (as expected^43^) following 18 hours culture in 1% O_2_ (Fig. 4B); again VEGF promoter activity was reduced following treatment with SEMA3F (44%, p < 0.05), but not to basal levels. To test the relative effect of SEMA3F on mTORC1/C2, we transiently co-transfected U87MG cells with our 2DAkt construct and the full length VEGF promoter reporter construct, and we cultured the cells in the absence or presence of SEMA3F. We found that SEMA3F failed to attenuate VEGF promoter activity in 2DAkt transfected cells following treatment with DFO (Fig. 4C). Finally, we measured the effect of SEMA3F on the secretion of VEGF into conditioned media (by ELISA) in the absence or presence of DFO. DFO markedly increased VEGF production (by 160% compared to untreated cells, p < 0.001 (data not shown) and inducible VEGF protein secretion was significantly reduced by either SEMA3F, rapamycin (for 18 hours to target mTORC1/C2) or by Torin 1 (Fig. 4D, p < 0.01). Concomitant treatment of the cells with rapamycin and SEMA3F failed to further suppress VEGF production, but the combination of SEMA3F and Torin 1 slightly (but significantly p < 0.05) inhibited VEGF levels as compared to SEMA3F or Torin 1 alone (Fig. 4D). Collectively, these findings indicate that SEMA3F suppresses inducible VEGF expression in part via the regulation of mTOR activity.

### SEMA3F inhibits U87MG tumor growth and angiogenesis *in vivo*

**Figure 5:**
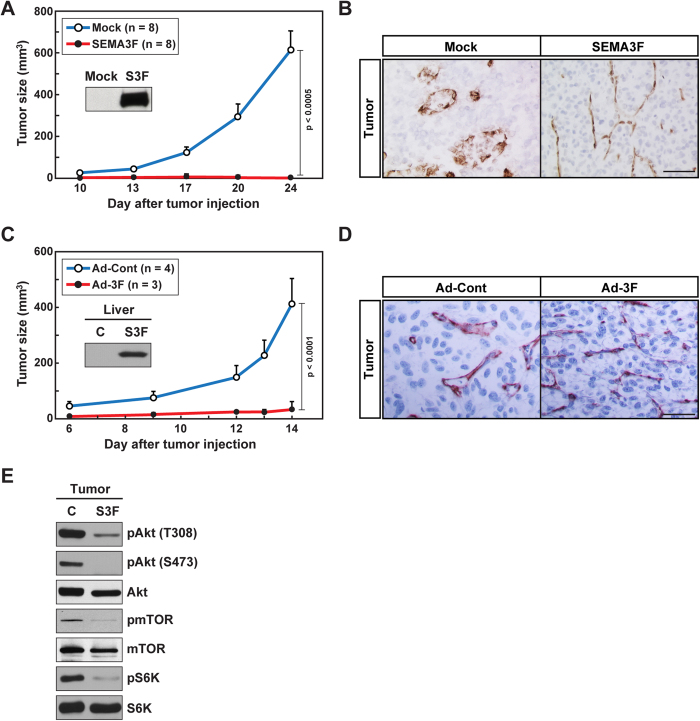
**SEMA3F inhibits human tumor growth in xenografts *in vivo*.** **A**, Parental U87MG cells (Mock)and human SEMA3F stable clones (S3F) were implanted into nude mice subcutaneously (1 × 10^6^ cells/injection). The insert shows Western blot analysis of SEMA3F expression in each cell line. Tumor size was measured using a standard calipers at the indicated time points. Numbers in parentheses represent the number of animals in each group. **B**, Representative immunohistochemical anti-CD31 staining of tumors harvested after 24 days. **C**, U87MG cells (1 × 10^6^ cells/injection) were administrated subcutaneously into nude mice. After 2 days, control (Ad-Cont) or human SEMA3F-His (Ad-3F)-recombinant adenovirus (1 × 10^9^ pfu) were injected intravenously via the tail vein. Tumor size was measured using a standard calipers at the indicated time points. Numbers in parentheses represent the number of animals in each group. Mice were sacrificed on day 14. The insert shows SEMA3F expression within the liver (on day 14) by Western blot analysis using an anti-His antibody. **D**, Representative immunohistochemical staining of tumors with anti-CD31. **E**, Western blot analysis of Akt/mTOR signaling pathway within tumor samples. Panels **B,D,E** are representative results of 3 independent experiments.

To next evaluate the *in vivo* relevance of our signaling studies, we examined the effect of SEMA3F on tumor growth in a well-established xenograft model^5^,^44^,^45^. In one approach, parental U87MG cells or U87MG cells that were engineered to constitutively overexpress SEMA3F were implanted subcutaneously into nude mice (1 × 10^6^/mouse); tumor size (mm^3^) was measured at the indicated time points over a period of 3 weeks. We found that tumor growth was essentially absent when SEMA3F-producing cells were implanted vs. parental cells (p < 0.0005, Fig. 5A). Furthermore, immunohistochemical analysis of CD31-expressing EC demonstrated numerous blood vessels in parental U87MG tumors (Fig. 5B); in contrast, capillaries within the U87MG/SEMA3F-derived tumors were constricted and were without discernable lumens (Fig. 5B).

Our second approach involved the injection of 1 × 10^6^ U87MG cells into the skin of nude mice, and after 2 days the mice received a single intravenous injection of adenovirus encoding human SEMA3F tagged with His (Ad-3F) or a control adenovirus (Ad-Cont). Injection of Ad-3F (1 × 10^9^ pfu) into mice did not result in any toxicity over a 30-day period; mice gained weight and typical behavior was normal. Western blot analysis showed that the administration of Ad-3F resulted in high levels of SEMA3F production in the liver (Fig. 5C), and by ELISA, SEMA3F levels were measureable in the serum. Circulating serum levels of SEMA3F protein peaked on day 8 following administration of adenovirus (day 10 post injection of tumor cells), and persisted until the end of the experiment on day 14 (average of 26 ng/ml, n = 4). Thus, this approach enables circulating SEMA3F production to begin at a time after tumor growth has been established in the mouse. We found that tumor volume reached 400 mm^3^ by day 14 in Ad-Cont-treated mice, whereas tumor growth was minimal over a 14 days period in mice injected with Ad-3F (p < 0.0001, Fig. 5C). By immunostaining, there was a striking collapsed phenotype of CD31-expressing capillaries within tumors harvested from Ad-3F-treated mice and most blood vessel lumens did not appear patent (Fig. 5D). Furthermore, by Western blot analysis, we found that pAkt, pmTOR and pS6K levels were suppressed in tumors following treatment with Ad-3F, as compared to Ad-Cont-treated mice (Fig. 5E). Thus, in two very different approaches, we find that SEMA3F administration (local and/or systemic) has similar anti-tumor and anti- angiogenesis effects by inhibiting the Akt/mTOR signaling pathway.

## Discussion

In these studies, we define SEMA3F as a potent mTOR inhibitor, and we show that its effect is mediated through the inhibition of PI-3K activity and the assembly of mTOR/rictor and mTOR/raptor complexes. We also find that its functional effect is mediated via interactions with the NRP2-Plexin A1 receptors. The regulation of mTOR by SEMA3F was found in several cell types, including T cells, endothelial cells and tumor cell lines, all of which are well established to utilize this signaling pathway for cellular activation, differentiation and proliferation. Our findings are suggestive that SEMA3F biology is of broad relevance in many physiological and pathological conditions, including cancer and diseases associated with chronic inflammation including allograft rejection.

While much is known about the intracellular regulation of mTORC1, relatively little is known about the upstream regulation of mTORC2 activity. In *C. elegans*, it has been shown that invertebrate semaphorin-plexin interactions reduce the association of TOR with rictor but promote TOR/raptor association, resulting in mRNA translation through the 4EBP-eIF4F pathway^14^. Our studies indicate that SEMA3F serves as a unique soluble ligand to selectively target mTORC2 activity in mammalian cells and thus elicit pro-resolution following cellular activation. For example, we transfected our NRP2-expressing cell lines with 2DAkt to activate mTORC1; in this model, we found that SEMA3F had minimal effects on the association between mTOR and raptor (data not shown) or the phosphorylation/activation of S6K and S6. In addition, SEMA3F responses were notably different than those observed following short time-course rapamycin treatment, which is known to primarily target mTORC1. In contrast, in several assays, we found that the inhibitory effects of SEMA3F on cellular activation and cytoskeletal collapse were primarily mediated through its effect on mTORC2 and were similar to those observed following treatment with Torin 1 (a pharmacological inhibitor of mTORC1/C2).

Importantly, we also found that SEMA3F reduced PI-3K activity which is reported to function in the activation of mTORC2^46^,^47^. It is thus likely that SEMA3F inactivates mTORC2 via upstream inhibition of PI-3K. Another related family member NRP1 binds and activates phosphatase and tensin homologue deleted on chromosome ten (PTEN)^7^, a negative regulator of PI-3K. We thus postulated that one mechanism may involve the recruitment of PTEN, which in turn serves as an intermediary to regulate PI-3K activity. Consistent with this possibility, we found that PTEN co-immunoprecipitated with NRP2 in human umbilical vein EC (HUVEC, Supplementary Fig. 4A). Moreover, following siRNA transfection and knockdown of Plexin A1, by immunoprecipitation, PTEN maintained association with NRP2 (Supplementary Fig. 4B). Furthermore, SEMA3F failed to inhibit pAkt expression following siRNA knockdown of PTEN in HUVEC (Supplementary Fig. 4C). These findings are most suggestive that the recruitment of PTEN to NRP2 is in part mechanistic for its regulatory effects on PI-3K/Akt/mTOR signaling.

However, U87MG, U251 and Jurkat cells are reported to be relatively PTEN deficient^48^,^49^,^50^ (see Supplementary Fig. 1C), suggesting that SEMA3F may also elicit its regulatory response(s) via PTEN-independent mechanisms. Indeed, as expected, PTEN failed to co-immunoprecipitate with NRP2 in U87MG cells (data not shown). To this end, we screened other adaptors with potential to mechanistically link NRP2 signals with PI-3K activity. These include GIPC1 (GAIP/RGS19-interacting protein, also known as neuropilin-interacting protein or synectin, Supplementary Fig. 4D)^51^,^52^,^53^, other GIPC family members^52^ and DEP domain containing mTOR interacting protein (DEPTOR)^32^,^54^. However, siRNA knockdown of these adaptors did not alter the regulatory effect of SEMA3F on pAkt expression (data not shown). We also evaluated crosstalk between the effects of SEMA3F on mTOR/Akt and MAPK signaling. The pharmacological MEK inhibitor U0126 did not modulate the regulatory effects of SEMA3F on levels of pAkt, indicating that the inhibitory effect of SEMA3F on Akt-mTOR signaling is MAPK-independent (Supplementary Fig. 4E). Thus, while SEMA3F-NRP2 interactions may recruit PTEN to regulate PI-3K and mTORC2 in primary cultures of normal cells, additional adaptors/kinases may also function in this response.

**Figure 6:**
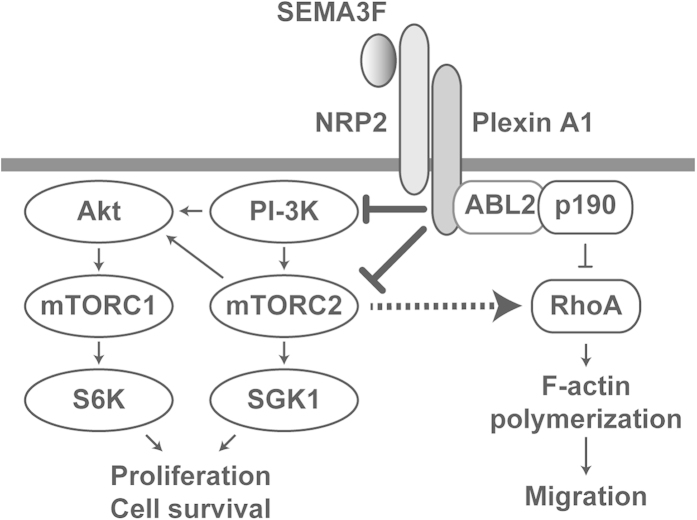
Schematic cartoon showing regulatory signaling pathways mediated by SEMA3F-NRP2/Plexin A1 interactions. SEMA3F binds to the NRP2-Plexin A1 complex and associates with PTEN to inactivate PI-3K and mTORC2/Akt-dependent signaling. Receptor-mediated signals may also inactivate mTORC2/Akt signaling via PTEN-independent mechanisms in tumor cell lines. Functionally, these regulatory/pro-resolution signals suppress cell proliferation, migration, cytoskeletal stress fiber rearrangement and cell survival. Our previous findings demonstrate that SEMA3F also inhibits cytoskeleton structure in part by inactivating RhoA through both the ABL2 kinase and p190RhoGAP^6^; the current studies show that the inactivation of RhoA and cytoskeletal stress fiber rearrangement is also mediated via the inhibition of mTORC2.

The semaphorin family of axonal guidance molecules, including SEMA3F, are well established to promote neuronal growth cone collapse that results from concomitant rearrangement of actin cytoskeletal stress fibers. SEMA3F is a potent inhibitor of tumor cell and EC adhesion, spreading and motility *in vitro* and *in vivo*^5^,^6^. In addition, SEMA3F does not induce apoptosis in U87MG cells within 24 hours^6^. In tumor and vascular endothelial cells, we previously observed that SEMA3F inactivates RhoA, thereby inhibiting cytoskeletal stress fiber formation^6^,^12^. In this report, we extend our signaling studies and demonstrate that mTORC2 is an intermediary in this response, and is indispensable for RhoA inactivation. For example, we show that SEMA3F treatment results in cell collapse following transfection of cells with mTOR (to induce mTORC1), suggesting that this effect is either mTOR-independent and/or is associated with targeting of mTORC2. Consistent with an effect on mTORC2, siRNA knockdown of rictor or the treatment of U87MG cells with the mTORC2 inhibitor Torin 1 consistently reduced the number of stress fibers as observed following treatment with SEMA3F. Importantly, we also noted that SEMA3F further reduced stress fibers in rictor-siRNA treated cells, indicating that SEMA3F likely inactivates RhoA in part via mTORC2 and in part via another mechanism such as the ABL2/p190RhoGAP pathway as we previously described^6^ (Fig. 6). Although mTORC2 is reported to interact with the Rho GTPase family and mediate F-actin cytoskeleton re-organization^18^,^55^, our new findings indicate that this effect can be targeted through stimulation of NRP2-induced signals.

We previously reported that mTORC2-dependent activation of Akt functions in the transcription of VEGF in endothelial cells^36^. VEGF functions as a pro-angiogenesis factor to augment tumor growth, and as a leukocyte chemoattractant in association with chronic inflammation^43^. Since SEMA3F targets mTORC2 activity, we postulated that it regulates the inducible expression of VEGF. Indeed, we found that SEMA3F markedly inhibits inducible VEGF expression via the inhibition of both mTORC2 and mTORC1. However, SEMA3F failed to inhibit the transactivation of VEGF following transfection of cells with the 2DAkt construct which induces mTORC1 activation. Also, treatment with either SEMA3F or rapamycin (long-term treatment to inhibit both mTORC1/C2) or the mTORC1/C2 inhibitor Torin 1 resulted in a similar level of inhibition of VEGF expression in U87MG cells. There was no additional inhibitory effect of combined SEMA3F and rapamycin, suggesting that SEMA3F and rapamycin target the same signaling pathway. However, surprisingly SEMA3F partially augmented the inhibitory effect of Torin 1 on VEGF expression. This may suggest that the Torin 1 dose used in these studies was not sufficient to completely inhibit mTORC2, or alternatively, it is possible that Torin 1 uncovers additional SEMA3F-elicited regulatory mechanism(s). Of note, SEMA3F alone fails to inhibit VEGF expression to basal levels, yet it has been previously reported to inhibit VEGF-induced proliferation of EC^56^. Nevertheless these new findings suggest that this anti-VEGF effect of SEMA3F may be most significant for its biological effects *in vivo*, such as our past^5^,^6^ and current observations on tumor growth inhibition.

Overexpression of SEMA3F in tumor cells, such as lung, brain and breast cancer cells was previously reported to inhibit tumor development in xenograft mouse models^5^,^13^,^44^,^45^,^57^. In these studies, we used an adenovirus to evaluate the effects of high levels of circulating SEMA3F protein on tumor growth and angiogenesis after tumors have developed. Most notably, circulating SEMA3F markedly inhibited tumor growth and all neoangiogenic blood vessels within the growing tumors had a dramatic collapsed phenotype, which is consistent with known SEMA3F effects on the cytoskeleton that we observed *in vitro*^6^. In addition, lysates of tumors from SEMA3F-treated mice showed diminished levels of pAkt, pmTOR and pS6K, which is consistent with its effects *in vitro*. Therefore, *in vivo* SEMA3F is likely to have an impact on tumor growth via both the suppression of VEGF secretion and direct inhibition of Akt-mTOR signaling. Also, the marked inhibition of tumor growth obtained by two different approaches (local overexpression by the tumor and by systemic administration) confirms that SEMA3F is a potent mTOR inhibitor *in vivo*.

In conclusion, these findings demonstrate that SEMA3F-NRP2 interactions inhibit intracellular PI-3K activity, mTORC2-dependent signaling, RhoA activity and cytoskeletal stress fiber formation. SEMA3F also inhibits the inducible expression of VEGF at both the transcriptional and protein level *in vitro*, and it has powerful anti-tumor effects *in vivo*. We conclude that SEMA3F is a secreted physiological mTOR inhibitor that functions to promote resolution following cellular activation. Our findings suggest that SEMA3F has therapeutic potential, not only in tumor biology, but also for instance to target chronic immune-mediated diseases, allograft rejection and/or angiogenesis related pathology.

## Methods

### Antibodies and reagents

The antibodies, rabbit monoclonal anti-phospho-Akt (Thr308) antibody (#2965); mouse monoclonal anti-phospho-Akt (Ser473) antibody (#4051); rabbit polyclonal anti-Akt antibody (#9272); rabbit polyclonal anti-phospho-Erk1/2 (Thr202/Tyr204) antibody (#9101); mouse monoclonal anti-Erk1/2 antibody (#4696); rabbit monoclonal anti-phospho-S6K (Thr389) antibody (#9234); rabbit monoclonal anti-S6K antibody (#2708); rabbit monoclonal anti-phospho-S6 (Ser235/236) antibody (#4856); mouse monoclonal anti-S6 antibody (#2317); rabbit monoclonal anti-phospho-mTOR (Ser2448) antibody (#5536); rabbit polyclonal anti-mTOR antibody (#2972); rabbit polyclonal anti-plexin A1 antibody (#3813); rabbit monoclonal anti-raptor antibody (#2280); rabbit monoclonal anti-RhoA antibody (#2117); rabbit monoclonal anti-PTEN antibody (#9188) were all purchased from Cell Signaling Technology (Danvers, MA). Goat polyclonal anti-phospho-SGK (S422, sc-16745); mouse monoclonal anti-NRP2 antibody (C-9, sc-13117); goat polyclonal anti-GIPC antibody (N-19, sc-9648) were purchased from Santa Cruz Biotechnology, Inc (Dallas, TX). Rabbit polyclonal anti-rictor antibody (A300-458A) was purchased from Bethyl Laboratories, Inc (Montgomery, TX), and mouse monoclonal anti-β-actin antibody (AC-15) was from Sigma-Aldrich (St. Louis, MO).

The VEGF-A (DVE00) ELISA kit was obtained from R&D Systems (Minneapolis, MN). The PI3-Kinase Activity ELISA (K-1000s) was purchased from Echelon Biosciences (Salt Lake City, UT). The mTOR inhibitors, rapamycin and Torin 1, were purchased from Lc Laboratories (Woburn, MA) and R&D Systems, respectively. Desferrioxamine (DFO) was purchased from Sigma-Aldrich, and the MEK inhibitor (U0126) was purchased from EMD-Millipore (Billerica, MA). Wild type (WT) mTOR vector was kindly provided to David M. Briscoe (by Dr. Robert T. Abraham, Pfizer Worldwide Research and Development, Oncology Research Unit)^58^. A VEGF promoter luciferase construct plasmid encoding the 2.6 kb full-length human VEGF was kindly provided by Dr. Debabrata Mukhopadhyay (Mayo Clinic, Minneapolis, MN)^59^. pGL4.74[hRluc/TK] vector (Promega Madison, WI) was used as an internal control in luciferase assay.

### Cell culture

U87MG and U251 human glioblastoma cells, kidney 293 cells and 293T cells, and Jurkat T lymphocytes were obtained from American Type Culture Collection (ATCC, Manassas, VA) and cultured in media containing 10% FBS (Denville Scientific, Inc., South Plainfield, NJ) and 1% L-glutamine/penicillin G/streptomycin sulfate (1% GPS, Life Technologies) as recommended. HUVECs were purchased from Lonza (Walkersville, MD) and cultured in EBM2 medium supplemented with EGM2 SingleQuot. Human melanocytes (HEMn-LP, Life Technologies) were maintained with Medium 254 supplemented with Human Melanocyte Growth Supplement (Life Technologies) in a 5% CO_2_ incubator at 37 °C. For all hypoxia experiments, cells were cultured in a hypoxic chamber (Heracell, Thermo Scientific, Hudson, NH) in 1% O_2_ at 37 °C.

### Human recombinant SEMA3F

A full-length, His-Myc-tagged human SEMA3F construct was transfected into 293T cells using FuGENE HD Transfection Reagent (Roche, Basel, Switzerland). SEMA3F secreted into culture medium was purified on HiTrap HP Chelating columns (GE Healthcare Bio-Sciences Corp., Pittsburgh, PA)^60^.

### Phospho-kinase array

The Human Phospho-Kinase Array Kit (ARY003) was obtained from R&D Systems. U87MG cells were treated with SEMA3F which was previously found to induce cytoskeletal collapse and inhibit RhoA activity^6^. We had previously treated U87MG cells with SEMA3F at 320 ng/ml which was found to induce morphological changes and inhibit cell migration in U87MG cell and HUVEC. Consistent with these results, we find that SEMA3F (even at the lowest concentration 200 ng/ml) inhibits pAkt and pS6K signaling (Supplementary Fig. 1D). However, in other cell types, we found this concentration to be suboptimal to suppress these signals. Thus, we optimized a concentration at 640 ng/ml to analyze SEMA3F signaling pathways. Cell lysates were collected at 30 minutes after SEMA3F treatment and the levels of phospho-proteins were analyzed with this array, according to the manufacturer’s instructions.

### Western blotting

Proteins within each sample were separated by SDS-PAGE and transferred to nitrocellulose membranes. The membranes were blocked with 4% skimmed milk in TBS-T (0.1% Tween 20 in tris-buffered saline [TBS]) for 30 minutes, followed by incubation with the primary antibody. After washing with TBS-T, membranes were incubated with the appropriate horseradish peroxidase-conjugated secondary antibody, and immunoreactivity was detected by using ECL detection reagents.

### Immunoprecipitation

Cell lysates were immunoprecipitated using an appropriate antibody at 4 °C overnight. Protein G-Sepharose 4 Fast Flow beads (GE Healthcare) were added to each sample, followed by mixing for 1 hour at 4 °C. The samples were dissolved in SDS sample buffer and boiled for 5 minutes.

### F-actin staining

Cells were fixed with 4% paraformaldehyde (PFA) followed by permeabilization with 0.2% Triton X-100 in PBS. F-actin and nuclei were stained with Alexa Fluor 488 phalloidin and Hoechst 33342, respectively. Confocal images from 3–5 areas of each culture were reviewed and stress fibers were counted in representative individual cells (~5/experiment) using standard methodology as described^6^.

### RhoA activity

RhoA activity assays were measured by using the RhoA activation assay kit based on rhotekin pull-down, according to the manufacturer’s instructions (Cytoskeleton, Denver, CO).

### RNA interference

Transfection of siRNA (20 nM) was performed with siLentFect Lipid Reagent (Bio-Rad, Hercules, CA), according to the manufacturer’s protocol. Control siRNA (Silencer Negative Control #2 siRNA) was purchased from Life Technologies. ON-TARGETplus Human NRP2 siRNA was purchased from Thermo Scientific (Hudson, NH), Plexin A1 (Hs_PLXNA1_3), rictor (Hs_RICTOR_5), PTEN (Hs_PTEN_6) and GIPC1 (Hs_RGS19IP1_1) siRNA from Qiagen (Valencia, CA), and Raptor siRNA (sc-44069) from Santa Cruz Biotechnology, Inc. (Dallas, TX). In general, siRNA transfection was performed for 48 hours prior to assays.

### Transfection and Luciferase assay

U87MG cells were transiently transfected with plasmid constructs (pcDNA3.1, WT mTOR, 2DAkt, VEGF luciferase reporter plasmid or pGL4.74[hRluc/TK]) as indicated using Lipofectamine 2000 reagent (Life Technologies) according to the manufacturer’s instructions. After 18 hours, cells were treated as outlined in each experimental design. VEGF promoter activity was analyzed using a Dual-Luciferase Reporter Assay System (Promega), and luciferase activity was normalized by Renilla luciferase as an internal control.

### Adenovirus

Recombinant control (#000047A) and human SEMA3F-His (#129755A) adenovirus were purchased from Applied Biological Materials, Inc. (Richmond, Canada). Each adenovirus was amplified with 293 cells and purified using the Fast Trap Adenovirus Purification and Concentration Kit (EMD-Millipore). The adenovirus titer was determined by Adeno-X™ Rapid Titer Kit (Clontech Laboratories, Inc., Mountain View, CA). We obtained each adenovirus at titers greater than 1 × 10^10^ pfu/ml.

### Tumor xenograft model

Parental U87MG cells or human SEMA3F stable U87MG clones (1 × 10^6^/injection) were administrated into nude mice (male, 8–10 weeks of age) subcutaneously. In one model, tumor size was measured every 3–4 days using a standard calipers. Mice were sacrificed on day 24 and tumors were removed. In a second model, parental U87MG cells were administrated into nude mice subcutaneously. In pilot studies, we injected adenovirus encoding SEMA3F (Ad-3F) or a control adenovirus (Ad-Cont) intravenously via the tail vein 3 days prior to tumor cell injection (1 × 10^6^ U87MG cells/injection); we observed that all tumors in the Ad-3F group failed to grow (data not shown). Thus, we revised our approach, and we delayed administration of Ad-3F until day 2 after the tumor injection, so that tumor growth was initiated prior to peak SEMA3F production in the circulation (~day 8–10 post administration, data not shown). Tumor size was measured every other day using a standard calipers, serum samples were collected from the tail vein at day 5 and 8 and mice were sacrificed on day 14 when the tumor, the liver and serum samples were collected. Production of SEMA3F was confirmed by Western blot analysis of liver with anti-His/anti-SEMA3F antibodies and by analysis of serum level of SEMA3F using the human SEMA3F ELISA kit (MBS454602) from MyBioSource (San Diego, CA). All animal studies were performed with approval in accordance with the guidelines of the Animal Care and Use Committee, Boston Children’s Hospital, Boston, MA.

### Immunohistochemistry

Paraffin-embedded sections were deparaffinized and activated with proteinase K (36 μg/ml) in 0.2 M Tris buffer (pH7.2) at 37 °C for 30 minutes and processed for immunohistochemical staining. Immunohistochemistry was performed with anti-mouse CD31 antibody (BD Biosciences, San Jose, CA), the VectaStain Kit (Vector, Burlingame, CA) and the Tyramide Signal Amplification (TSA) Biotin system (NEN Life Science Products, Boston, MA), according to the manufacturer’s instructions.

### Statistical analysis

All assays were independently performed at least three times. The results are represented as mean ± standard deviation (SD). Groups were compared using the Student’s t test and p values < 0.05 were considered statistically significant.

## Additional Information

**How to cite this article**: Nakayama, H. *et al.* Regulation of mTOR Signaling by Semaphorin 3F-Neuropilin 2 Interactions *in vitro* and *in vivo*. *Sci. Rep.* **5**, 11789; doi: 10.1038/srep11789 (2015).

## Supplementary Material

Supplementary Information

Supplementary Dataset
