# Supplementary material for: Regulation of mTOR Signaling by Semaphorin 3F-Neuropilin 2 Interactions In Vitro and In Vivo
Source: Sci Rep. 2015 Jul 9;5:11789. doi: 10.1038/srep11789 (PMC4496725; doi:10.1038/srep11789)

**REGULATION OF MTOR SIGNALING BY SEMAPHORIN 3F-NEUROPILIN 2 INTERACTIONS IN VITRO AND IN VIVO**

**Authors:** Hironao Nakayama^1,2,3,4^, Sarah Bruneau^2,5^, Nora Kochupurakkal^2,5^,

Silvia Coma^1,3^, David M. Briscoe^2,5,†,*^ and Michael Klagsbrun^1,3,6,†^

**Affiliations:**

^1^Vascular Biology Program, Boston Children’s Hospital and Harvard Medical School, 300 Longwood Avenue, Boston, MA 02115.

^2^Transplant Research Program, Boston Children’s Hospital and Harvard Medical School, 300 Longwood Avenue, Boston, MA 02115.

^3^Department of Surgery, Boston Children’s Hospital and Harvard Medical School, 300 Longwood Avenue, Boston, MA 02115.

^4^Division of Cell Growth and Tumor Regulation, Proteo-Science Center, Ehime University, Toon, Ehime 791-0295, Japan.

^5^Department of Pediatrics, Boston Children’s Hospital and Harvard Medical School, 300 Longwood Avenue, Boston, MA 02115.

^6^Department of Pathology, Boston Children’s Hospital and Harvard Medical School, 300 Longwood Avenue, Boston, MA 02115.

^†^David M. Briscoe and Michael Klagsbrun served as co-senior authors.

^*^Corresponding author. David M. Briscoe MD, Transplant Research Program, Boston Children’s Hospital, 300 Longwood Avenue, Boston, MA 02115. Tel 617-919-2992. Fax 617-730-0130. Email: david.briscoe@childrens.harvard.edu

**Supplementary Information**

Supplementary Fig. 1. Analysis of intracellular signaling pathway regulated by SEMA3F.

Supplementary Fig. 2. The effect of SEMA3F on mTORC2.

Supplementary Fig. 3. The low magnification images of the SEMA3F collapse assay.

Supplementary Fig. 4. Analysis of intracellular signaling pathway regulated by SEMA3F.

Supplementary Table 1. The dot intensity of phosphoprotein kinase antibody array.

**Supplementary Figures**

**SUPPLEMENTARY FIGURE 1**. Analysis of intracellular signaling pathway regulated by SEMA3F. *A,* U87MG cells were treated with SEMA3F or PBS for 60 minutes and expression of pAkt, pmTOR and pS6K were analyzed by Western blot. *B,* U87MG cells were transfected with control or Plexin A1-specific siRNA (20 nM). After 48 hours, cells were treated with SEMA3F (640 ng/ml) for 30 and 60 minutes, and were analyzed by Western blot. *C,* NRP2 and Plexin A1 expression were analyzed by Western blot with multiple cell lines. *D,* Multiple NRP2-expressing cell lines were treated with SEMA3F for 30 minutes and were analyzed by Western blot. All data presented are representative of 3 independent experiments.

**SUPPLEMENTARY FIGURE 2**. Analysis of the effect of SEMA3F on mTORC2 activity. *A,* U87MG cells were transiently transfected with a pcDNA3.1 empty vector or with constitutively active Akt (2DAkt). Cells were treated with SEMA3F (640 ng/ml) and lysates were analyzed by Western blot. *B,* U87MG cells were transiently transfected with a pcDNA3.1 empty vector or with 2DAkt. Cells were treated with SEMA3F (640 ng/ml) for 30 minutes and were subjected to immunoprecipitation and Western blot analysis with anti-mTOR, and anti-rictor as illustrated.

**SUPPLEMENTARY FIGURE 3**. Low magnification images of the SEMA3F collapse assay illustrated in Fig. 3A. U87MG cells were treated with SEMA3F (640 ng/ml), rapamycin (10 nM) or Torin 1 (10 nM) for 30 minutes. Cells were stained with Alexa Fluor 488 phalloidin and Hoechst 33342.

**SUPPLEMENTARY FIGURE 4**. *A,* HUVEC were treated with SEMA3F (1800 ng/ml) for 30 minutes and were subjected to immunoprecipitation and Western blot analyses with anti-NRP2 and -PTEN as illustrated. *B,* HUVEC were transfected with control-, or Plexin A1-specific siRNAs (20 nM), prior to SEMA3F treatment (1800 ng/ml); lysates were subjected to immunoprecipitation and Western blot analyses with anti-NRP2 and anti-PTEN as illustrated. *C,* HUVEC were transfected with control-, or PTEN-specific siRNAs (20 nM), prior to SEMA3F treatment (1800 ng/ml); lysates were analyzed by Western blot. *D,* U87MG cells were transfected with control or GIPC1-specific siRNA (20 nM). After 48 hours, cells were treated with SEMA3F (200, 600, 1800 ng/ml, from left to right) for 30 minutes, and were analyzed by Western blot. *E,* U87MG cells were treated with U0126 (10 μM) for 30 minutes prior to combination with 30 minutes and 60 minutes of SEMA3F (640 ng/ml). Akt and MAPK signaling was analyzed by Western blot. All data presented are representative of 3 independent experiments.

**SUPPLEMENTARY TABLE 1**. The intensity of each dot/phosphoprotein was measured using Image J software.

**SUPPLEMENTARY FIGURE 1**.


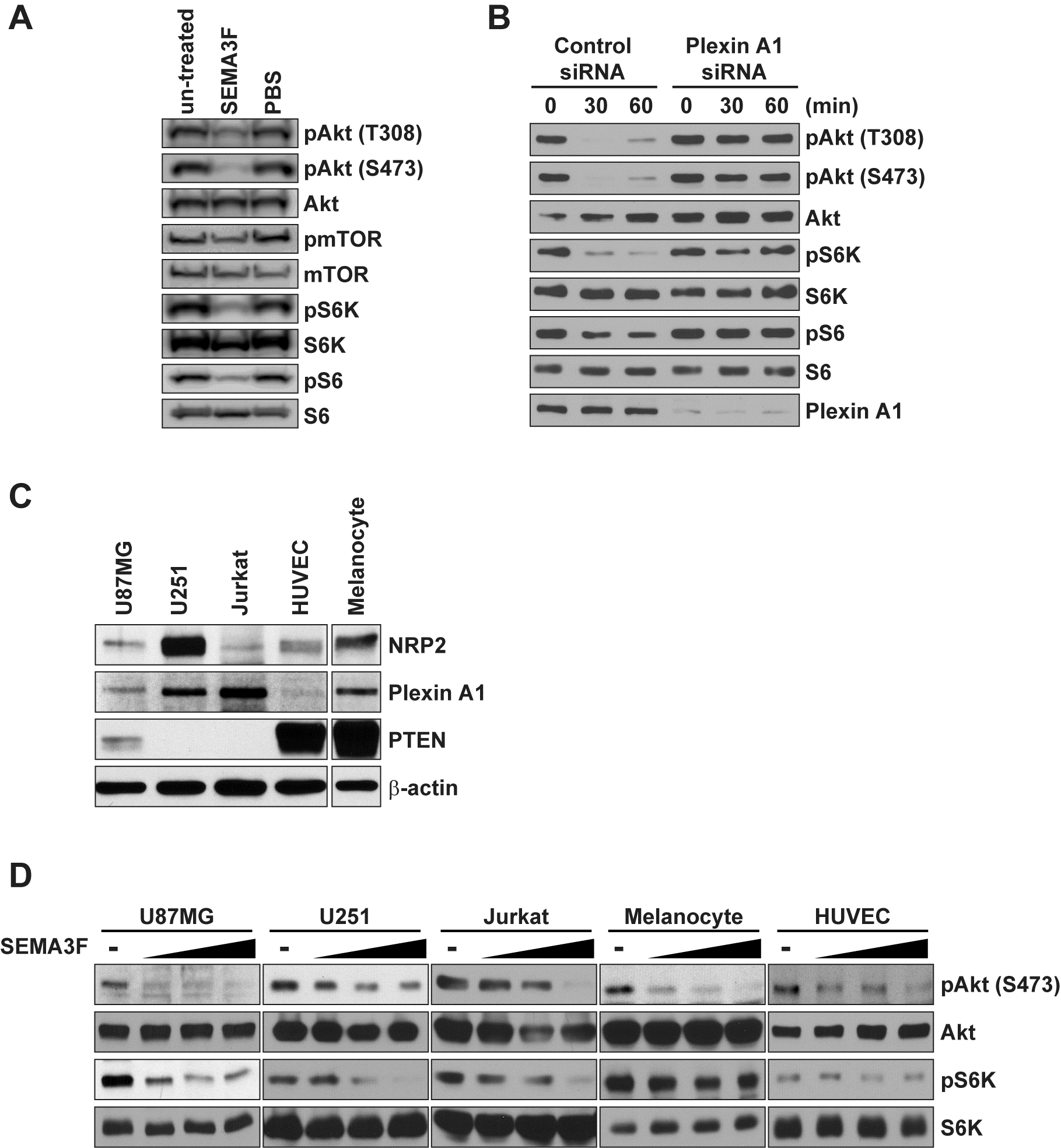


**SUPPLEMENTARY FIGURE 2**.


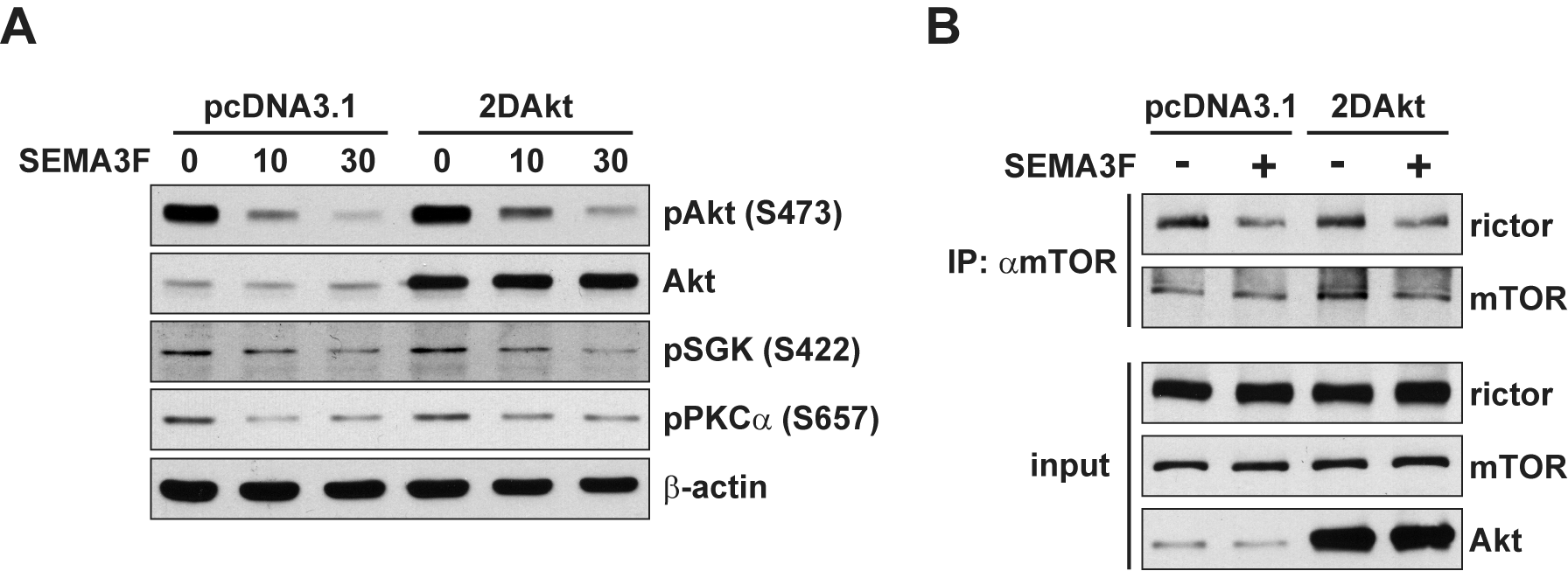


**SUPPLEMENTARY FIGURE 3**.


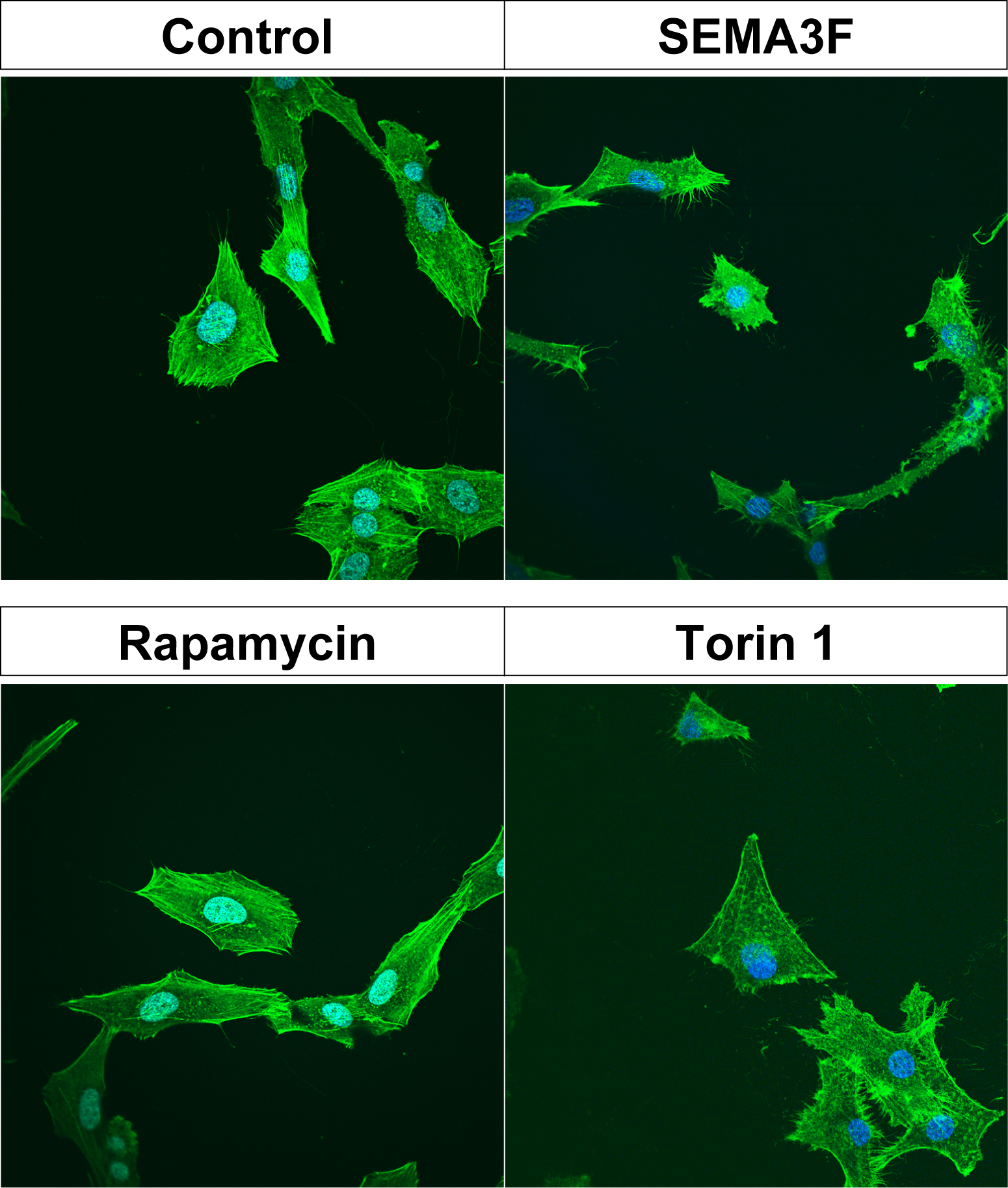


**SUPPLEMENTARY FIGURE 4**.


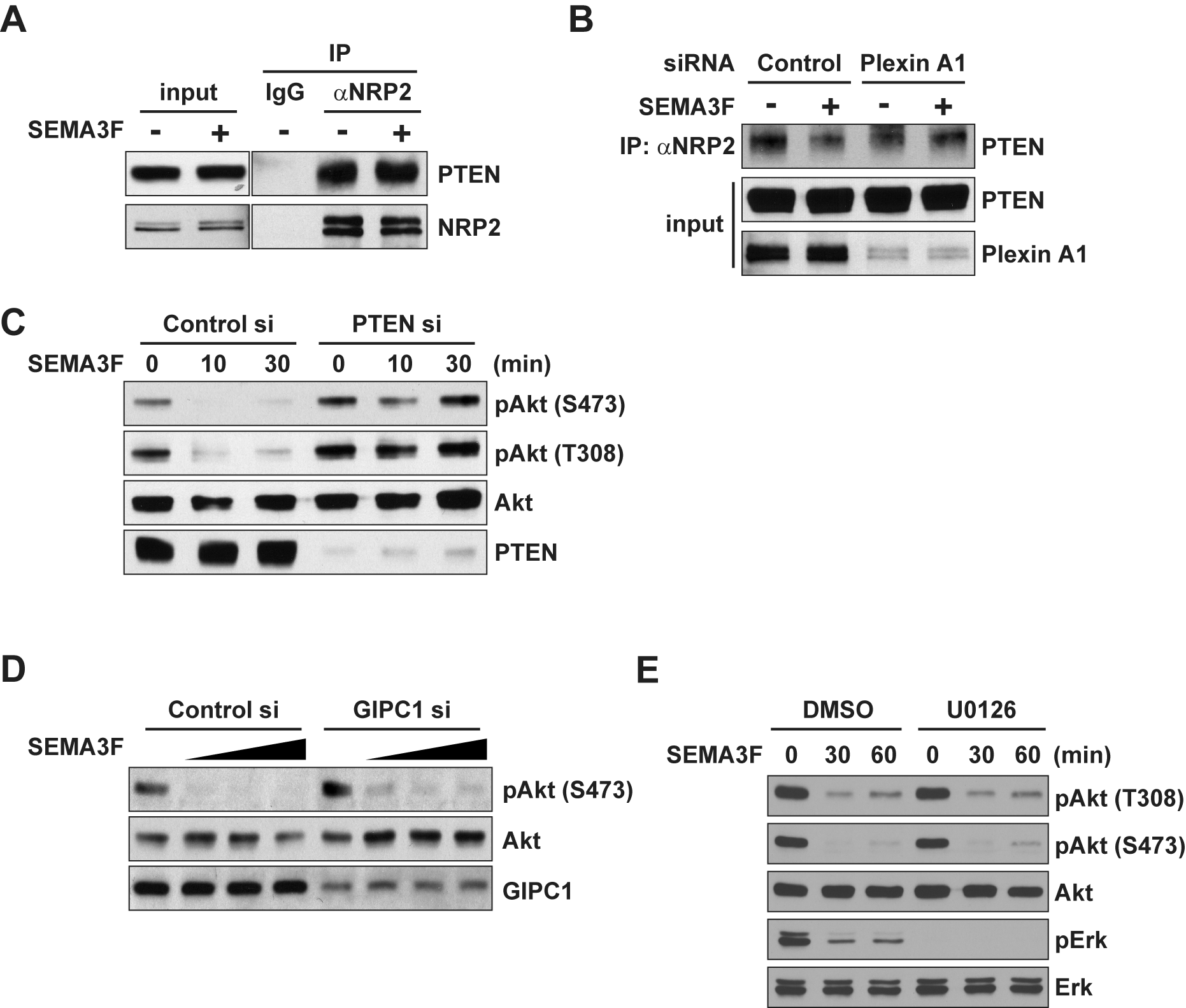


**SUPPLEMENTARY TABLE 1**.


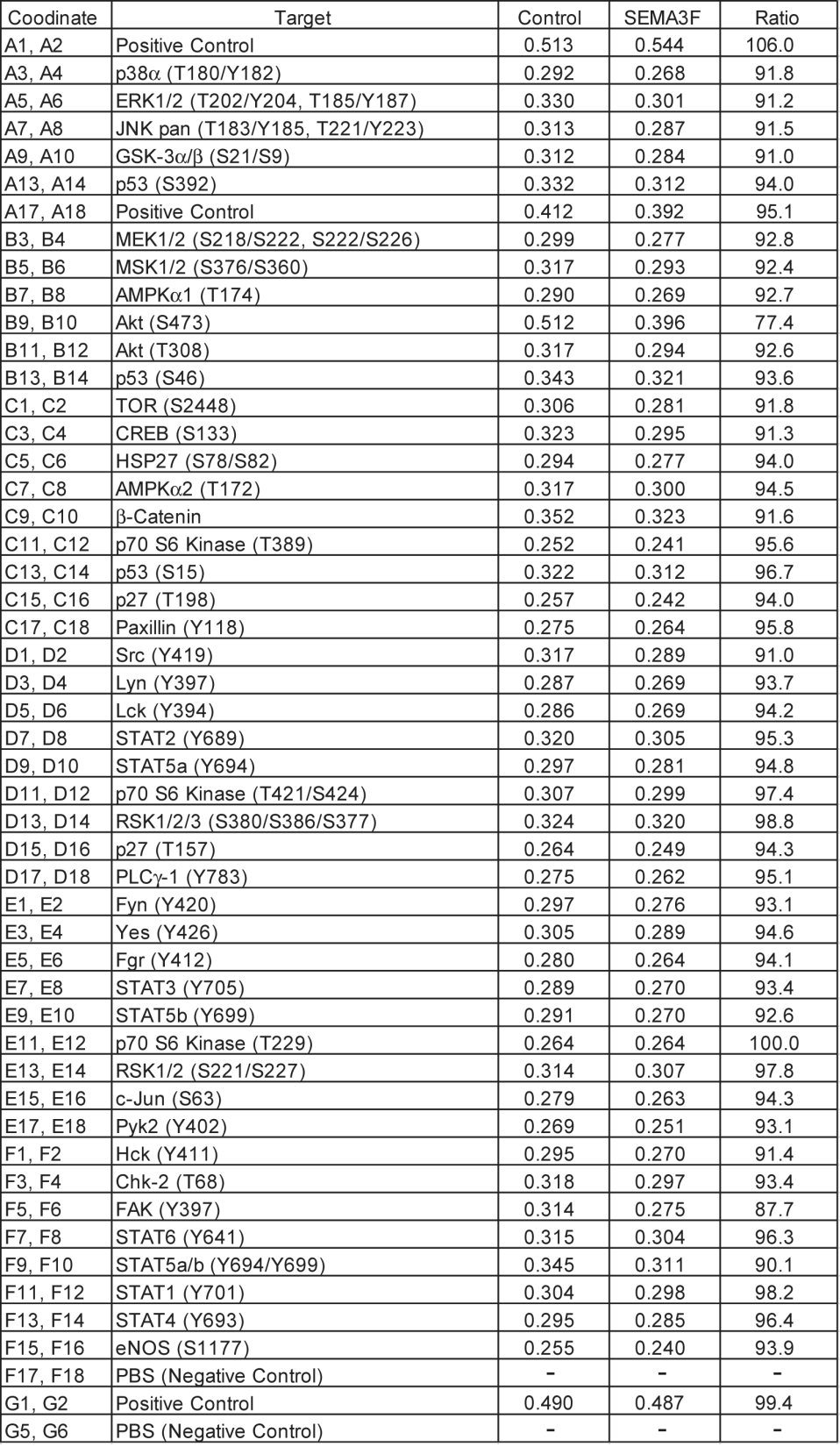

Supplement: Supplementary Information [file srep11789-s1.doc]
